# Resting-state functional connectivity in an auditory network differs between aspiring professional and amateur musicians and correlates with performance

**DOI:** 10.1007/s00429-023-02711-1

**Published:** 2023-10-04

**Authors:** Eleftheria Papadaki, Theodoros Koustakas, André Werner, Ulman Lindenberger, Simone Kühn, Elisabeth Wenger

**Affiliations:** 1 Center for Lifespan Psychology, Max Planck Institute for Human Development, Lentzeallee 94, 14195 Berlin, Germany; 2 International Max Planck Research School on the Life Course (LIFE), Berlin, Germany; 3 Max Planck UCL Centre for Computational Psychiatry and Ageing Research, Berlin, Germany, London, UK; 4 Lise Meitner Group for Environmental Neuroscience, Max Planck Institute for Human Development, Berlin, Germany; 5 Neuronal Plasticity Working Group, Department of Psychiatry and Psychotherapy, University Medical Center Hamburg-Eppendorf, Hamburg, Germany

**Keywords:** Resting-state fMRI, Auditory plasticity, Musicians, Graph theoretical measures, Interval identification

## Abstract

Auditory experience-dependent plasticity is often studied in the domain of musical expertise. Available evidence suggests that years of musical practice are associated with structural and functional changes in auditory cortex and related brain regions. Resting-state functional magnetic resonance imaging (MRI) can be used to investigate neural correlates of musical training and expertise beyond specific task influences. Here, we compared two groups of musicians with varying expertise: 24 aspiring professional musicians preparing for their entrance exam at Universities of Arts versus 17 amateur musicians without any such aspirations but who also performed music on a regular basis. We used an interval recognition task to define task-relevant brain regions and computed functional connectivity and graph-theoretical measures in this network on separately acquired resting-state data. Aspiring professionals performed significantly better on all behavioral indicators including interval recognition and also showed significantly greater network strength and global efficiency than amateur musicians. Critically, both average network strength and global efficiency were correlated with interval recognition task performance assessed in the scanner, and with an additional measure of interval identification ability. These findings demonstrate that task-informed resting-state fMRI can capture connectivity differences that correspond to expertise-related differences in behavior.

**Supplementary Information:**

The online version contains supplementary material available at 10.1007/s00429-023-02711-1.

## Introduction

Musicians have been a favored group in studies investigating experience-dependent plasticity and the neural correlates of expertise. The years-long intensive training that musicians undergo, often beginning at a very young age, puts great demands not only on specific brain regions in the auditory and motor cortex but also on multisensory and higher order cognitive-processing brain regions (Jäncke 2009). Such high demands constitute an ideal condition for triggering brain plasticity, manifested as alterations in brain structure and function in an effort to respond to the challenges posed (Lövdén et al. 2010).

Musicians, when compared to non-musicians, exhibit larger volumes in primary auditory cortex residing on Heschl’s gyrus, corresponding to differences in neurophysiological responses and musical aptitude (Schneider et al. 2002, 2005). Further differences in volume and cortical thickness in grey matter structure are reported in regions of secondary auditory cortex, motor and visuo-spatial processing as well as in frontal regions (Bermudez and Zatorre 2005; Gaser and Schlaug 2003; Palomar-García et al. 2017; Wenger et al. 2021). Differences are also found in white matter architecture and in structural connectivity of the white matter tracts (Abdul-Kareem et al. 2011; Leipold et al. 2021; Schmithorst and Wilke 2002) and in brain activation, during a variety of music-related tasks (Bangert et al. 2006; Bianchi et al. 2017; Limb et al. 2006) and during listening to music (Angulo-Perkins et al. 2014).

Interestingly, manifestations of brain plasticity have not only been investigated in the comparison of musicians versus non-musicians, but also in relation to different levels of musical expertise. In this perspective, musical expertise forms more of a continuum and the contribution of important factors, such as duration of training, intensity of training and overall intentions in music engagement, which relate to changes in brain structure and function, can be better understood. Differing levels of expertise, that is, professional, amateur, and non-musicians) actually appear distinct not only in behavioral measures but also in neural substrates: differences in grey matter volumes between professional and amateur musicians have been reported in motor, auditory and visuospatial regions as a result of practice intensity (Gaser and Schlaug 2003). In a study with a sample of professional, amateur and non-musicians, grey matter volume and neurophysiological responses from the Heschl’s gyrus were reported to be modulated by the level of expertise of each group, with amateur musicians being the intermediate between the other two groups (Schneider et al. 2002). In a series of very interesting studies investigating the neural correlates of different levels of expertise using tonal sequences containing different degrees of structural irregularities at their ending, gradual changes in the response amplitudes using fMRI were observed as a function of expertise level (James et al. 2017). In addition, a stepwise modulation of brain responses by expertise level in a frontoparietal network was visible, related also to working memory and attention processes, with overall brain activation of amateurs being intermediate between the other two groups, and partly overlapping with the responses of the professional's group (Oechslin et al. 2013). Stepwise increases in grey matter density were also reported in auditory and cognitive regions (James et al. 2014), and white matter tract consistency was also differentiated among the three groups, with increasing consistency corresponding to higher expertise level (Oechslin et al. 2018).

This multitude of plasticity manifestations in cross-sectional and longitudinal studies are complemented by studies examining the factors of predispositions manifested as different conditions in brain function and anatomy (Zatorre 2013) as well as of genetic differences predisposing individuals to successfully engage in music training (Ullén et al. 2016). Indeed, the amount of music practice has been found to be highly heritable, and associations between musical practice and musical aptitude are highly correlated with genetic differences (Mosing et al. 2014). However, the causal effects of training on changes in brain function and anatomy cannot be refuted, especially under the light of evidence concerning samples of monozygotic twins (de Manzano and Ullén 2018).

In the last years, resting-state functional magnetic resonance imaging (fMRI), capturing the intrinsic low-frequency fluctuations of brain activity exhibiting temporal and spatial organization (Raichle 2015) has established that the brain’s functional network architecture during task performance is actually predominantly sculptured by an intrinsic network architecture that is also present during rest (Cole et al. 2014, 2016). The intrinsic architecture has been related to various aspects of cognitive, social and emotional processes as well as to personality traits (Liégeois et al. 2019). It is regularly included in studies aiming at relating measures of functional organization and graph theoretical analysis to learning and performance in tasks targeting a variety of domains, including attention (Rosenberg et al. 2015), working memory (Hampson et al. 2006), memory consolidation (Collins and Dickerson 2019; Meskaldji et al. 2016), perception (Baldassarre et al. 2012), learning (Lumaca et al. 2019; Ventura-Campos et al. 2013) and motor skill acquisition (Bassett et al. 2011, 2015).

Measures of resting-state fMRI also have been used in the context of musical learning and expertise, complementing and extending findings from task-fMRI studies by capturing alterations in intrinsic brain organization. Musical expertise is reflected in interhemispheric and intrahemispheric connectivity patterns of functional networks (Leipold et al. 2021). Often, increased resting-state functional connectivity in musicians compared to non-musicians has been reported, primarily concerning the connections between regions of bilateral auditory cortices with the premotor, supramarginal and orbitofrontal regions (Fauvel et al. 2014; Luo et al. 2012; Palomar-García et al. 2017). Apart from regions specifically relating to the perception and execution of music, studies also suggest that musicianship is characterized by altered functional connectivity, both static and dynamic, between brain regions across the entire brain, including also multisensory regions and regions of various cognitive functions, such as memory, language and attention (Hou and Chen 2021; Hou et al. 2015; Luo et al. 2012), as well as higher order associative regions, such as the insula, potentially facilitating integration of multisensory information (Zamorano et al. 2017).

With the present study, we set out to investigate whether aspiring professional musicians differ in terms of their resting-state functional connectivity of an auditory network involved in interval recognition in comparison with amateur musicians, even though both groups have comparable years of playing an instrument. An interval is the definition of the distance between any two frequencies. In the European musical tradition since the first half of the 18th Century, on which this study is based, the pitch continuum is divided into discrete steps on a logarithmic scale (thus, 110–220 Hz and 220–440 Hz are both described as the same interval—an octave). The semi-tone or half-step is the smallest unit of measure: any interval can be described as a sum of semi-tones. Traditionally, intervals are described as a combination of size (2nd, 3rd, 4th, etc.) and quality (major, minor, perfect, diminished, augmented) based on their roles within the tonal system. Interval perception, both as the perception of pitch relations between tones of a chord and as the pitch relation of temporally sequential tones, lies at the core of tonal processing. Tonal processing includes the perception of the arrangement of pitches and chords around the tonal center, the first note of the scale, and their perceived hierarchical relations, stabilities, attractions and directionalities, within the context of the scales (ordered sequences of notes) they evoke (Zatorre 2003). An extensive amount of research has established that processing of acoustic information begins early in the auditory pathway, with the brainstem as a crucial layover in pitch perception before the primary auditory cortex takes over to transform the acoustic features into percepts (Koelsch 2011). From there on, processing in the auditory cortex appears to follow a hierarchical organization, beginning in the primary auditory cortex in Heschl’s gyrus, crucial for pitch perception and discrimination, and extending both anterolaterally and posteriorly with increasing features’ complexity (Chevillet et al. 2011; Peretz and Zatorre 2005). Next, secondary auditory cortices are consistently reported as crucial in perceptual analysis of tonal information, with both anterior and posterior parts of the superior temporal gyrus, the superior temporal sulcus, the planum polare, the planum temporale, being related to processing pitch height differences (Peretz and Zatorre 2005), in categorical pitch perception (Lee et al. 2011), as well as in consonance and dissonance processing (Bidelman and Grall 2014). Regions in posterior Superior Temporal Gyrus and frontal regions are repeatedly reported as supporting tonal processing with working memory and attentional mechanisms, with right inferior lateral frontal areas reported as important for maintenance of tonal information (Janata et al. 2002; King et al. 2018; Nolden et al. 2013).

To investigate whether resting-state functional organization can be an indicator of performance and a neural correlate of musical expertise in interval recognition, we utilized an fMRI task to localize regions in the auditory cortex and beyond, constituting a network specific to listening to and recognizing auditorily presented intervals. We examined the architecture of this network in resting-state using graph-theoretical measures and related it to performance in the intervals task as well as performance in another behavioral measure reflecting musical expertise. We expected that the identified network would include parts of the auditory network, prominently the primary auditory cortex and adjacent regions of the secondary auditory cortex, located bilaterally on the superior temporal gyri. We hypothesized that the two groups of the study, aspiring professional musicians and amateur musicians, would differ in terms of network strength and global efficiency. In addition, we hypothesized that stronger functional connectivity in the identified network, reflected in the graph measure of network strength, and more efficient within-network communication, captured by global efficiency, would correlate with better performance in the interval recognition task and with relevant parts of another behavioral assessment of musical expertise.

## Materials and methods

### Participants

We recruited 41 participants between 18 and 31 years of age (*M*_age_ = 22.35, SD = 3.63, 15 female). They were recruited through flyers, mailing lists, project presentations in music schools, and word-of-mouth recommendation in Berlin, Germany. Twenty-four of these individuals were in the process of preparing for the entrance exam for a music conservatory. Seventeen individuals were amateur musicians who were actively performing music in everyday life. All participants either sang or played at least one primary instrument, and had at least five or more years of experience singing or playing the respective instrument. Information on the primary instruments reported by participants in both groups can be found in Table 1 of supplementary material and a summary of the following information on sample characteristics can be found in Table 1. Years of singing or playing a primary instrument were comparable across the two groups, *t*(38) < 1, *p* = 0.68( amateur musicians: *M*_year_ = 12.74, SD = 5.97; aspiring professional musicians: *M*_year_ = 12.04, SD = 4.56; one participant in the aspiring professional group did not provide information about his or her primary instrument or years of playing). However, participants in the two groups differed in the daily amount of practice dedicated to instrument playing (*t*(39) = 3.7, *p* = 0.001, amateur musicians; *M*_hours_ = 1.2, SD = 0.8; aspiring professional musicians* M*_hours_ = 2.6, SD = 1.4) and to music theory learning (*t*(39) = 4.91, *p* = 0.001, amateur musicians; *M*_hours_ = 0.2, SD = 0.3; aspiring professional musicians* M*_hours_ = 1.4, SD = 0.6). Therefore, our sample comprises two groups of people who have been musically engaged for approximately the same amount of time. A decisive difference lies in the intensity of the training given the different intentions in their musical practice, with aspiring professional musicians undergoing intensive both practical and theoretical learning with their respective musical instruments to be accepted for music university programs. It is, therefore, not simply the mere amount of time of engagement with music that is characterizing different levels of expertise but rather the intensity of this engagement and the motivation behind it given the professional intention.

**Table 1:** Summary table of sample characteristics regarding age, years of engagement with primary instrument or voice training, daily amount of primary instrument practice, daily amount of music theoretical learning and handedness (for 5 participants there are no handedness information)

	Age (years)		Music learning primary instrument-voice (years)		Instrument practice (daily hours)		Music theory learning (daily hours)		Handedness	
	*M*	SD	*M*	SD	*M*	SD	*M*	SD	Left	Right
Aspiring professionals	21.92	3.72	12.04	4.56	2.6	1.4	1.4	0.6	1	20
Amateur musicians	23	3.5	12.04	5.97	1.2	0.8	0.2	0.3	1	14

Participants of both groups did not differ with respect to age, *t*(39) < – 1.05, *p* = 0.30 (amateur musicians; *M*_age_ = 23.00, SD = 3.50, 8 female; aspiring professional musicians* M*_age_ = 21.92, SD = 3.72, 7 female). Regarding handedness, 33 participants were right-handed, 2 were left-handed (one in the group of aspiring professionals and one in the group of amateur musicians) and for 5 participants (3 in the group of aspiring professionals and 2 in the group of amateur musicians) there was no report on their handedness. All participants had normal hearing, did not have any metallic implants, and had not had any psychiatric diagnosis.

The experiment reported here was part of a larger longitudinal study, including behavioral testing (described in more detail in Lin et al. 2021) as well as structural and functional MRI (see also Wenger et al. 2021, for longitudinal structural changes). Participants were paid up to 200€ for completion of the whole study (including up to 5 measurement time points with 1.5 h of MRI and 1.5 h of behavioral testing). The ethical board of the DGPs (Ethikkommission der Deutschen Gesellschaft für Psychologie) approved the study, and written consent of all participants was obtained prior to investigation.

### Behavioral measure Berlin Gehoerbildung Scale (BGS)

Participants’ level of music expertise was measured by the Berlin Gehoerbildung Scale (BGS, Lin et al. 2021). The BGS was designed by André Werner, a composer and collaborator of this study. The BGS aims at assessing various aspects of music expertise within the tradition of western art music and it is informed by music theory and uses a variety of testing methods in the ear-training tradition. The BGS requires listening to musical recordings, and the use of musical notation. It taps into various aspects of knowledge and skill in ear training and music theory, including intervals, scales, dictation (translation of chord progressions, melodies, rhythm into notation), rhythm, harmony, identifying deviations in music excerpts, and instrument recognition. It requires formal music education and training and is designed to assess the upper end of music achievement. The BGS consists of four factor-analytically validated scales, namely, Intervals and Scales, Dictation, Chords and Cadences, and Complex Listening, which together form a second-order factor of general music expertise. For the purpose of this study, we focused on the second-order scale of general music expertise, and first-order scale Intervals and Scales, which can be assumed to assess the same ability as the fMRI interval recognition task, and which comprises four items: naming intervals, notating intervals, naming scales and naming and notating scales (for more information, see Lin et al. 2021).

### fMRI interval recognition task

During the fMRI task, participants had to recognize the musical interval characterizing two tones. All the intervals presented are in accordance with the European/western traditional music in educational practice. On each trial, after hearing two tones that were either presented successively or simultaneously, participants had to choose among four options presented on the screen and indicate the correct interval label. The stimuli were recorded piano tones from a simulation program and had a standard duration of 1600 ms. After the presentation of the tones, there was a random jitter between 1.5 and 3 s, after which the response screen appeared. As soon as participants responded via a button press (or after a maximum of 20 s), there was an inter-stimulus interval of 1 s and a jitter between 1.5 and 3 s, after which the next trial started. Within a total task time ranging up to 20 min, 140 intervals were presented.

### MRI data acquisition

Magnetic resonance images were collected on a Siemens Tim Trio 3 T MR scanner (Erlangen, Germany) with a standard 12-channel head coil. For the structural images, a three-dimensional T1-weighted magnetization prepared gradient-echo sequence (MPRAGE) was used (TR = 2500 ms, TE = 4.77 ms, TI = 1100 ms, flip angle = 7°, bandwidth = 140 Hz/pixel, acquisition matrix = 256 × 256 × 192 mm^3^, isometric voxel size = 1 mm^3^). After that, an 8-min resting-state acquisition followed, while participants had their eyes open and were looking at a fixation cross, using a T2*-weighted EPI sequence sensitive to Blood Oxygenation Level Dependent (BOLD) contrast (TR = 2000 ms, TE = 30 ms, FOV = 216 × 216 × 129 mm^3^, flip angle = 80°, slice thickness 3.0 mm, distance factor = 20%, voxel size = 3 mm^3^, 36 axial slices, using GRAPPA acceleration factor 2). Following an auditory oddball task that is not part of the present study, the intervals task was acquired using the same T2*-weighted EPI sequence as described above. All slices were acquired in an interleaved fashion, aligned to genu splenium of the corpus callosum.

### Behavioral data analysis

*BGS.* We formed unit-weighted *z*-scores for the first-order scale Intervals and Scales by calculating the average of the four *z*-transformed items belonging to this subscale, and the second-order scale of general music expertise by calculating the average of all *z*-transformed subscales. These unit-weighted *z*-scores were subsequently submitted to independent samples *t* tests to test for group differences between aspiring professionals and amateur musicians.

*fMRI intervals recognition task* Performance on the intervals task was calculated for each participant as the percent of correct responses, that is task accuracy, using R (R Core Team 2021). As the data were not normally distributed and professional musicians showed a ceiling effect, we squared-root transformed the data and used a Mann–Whitney *U* test for independent samples to analyze group differences in task accuracy between aspiring professional and amateur musicians. In addition, we calculated the reaction times for each participant using the median across trials and we computed group differences between aspiring professional and amateur musicians in reaction times using a Mann–Whitney *U* test for independent samples, as the values were not normally distributed.

### fMRI data analysis

#### Preprocessing

Before starting with the MRI analysis, the acquired structural, task and rest data were structured according to the Brain Imaging Data Structure (BIDS) specifications (Gorgolewski et al. 2016). Data preprocessing of the task fMRI and rest fMRI data was performed using the fMRIPrep toolbox°20.2.0 (Esteban et al. 2019) with the default processing steps utilizing the software packages FSL, FreeSurfer, ANTs, and AFNI. For further details on each preprocessing step in fMRIprep, please refer to the online documentation under https://fmriprep.org/en/stable/. Briefly, a reference volume and its skull-stripped version were first generated. The BOLD reference image was then co-registered to the T_1_-weighted anatomical reference. Head-motion parameters with respect to the BOLD reference (transformation matrices, and six corresponding rotation and translation parameters) were estimated before any spatiotemporal filtering. The BOLD runs were then slice-time corrected and finally resampled into MNI152NLin2009cAsym standard space with a voxel size of 3 mm × 3 mm × 3 mm.

Several confounding time-series were calculated during preprocessing: framewise displacement (FD), Delta VARiation Signal (DVARS), and global signals were extracted for cerebrospinal fluid, white matter, and whole-brain masks, which were later used as nuisance regressors. In addition, a set of physiological regressors were extracted to allow for component-based noise correction (CompCor, Behzadi et al. 2007)*.* No individuals had to be excluded due to motion (no image exceeded 0.3 mm average FD).

The task fMRI data were then spatially smoothed with a 6 mm full-width half-maximum (FWHM) isotropic Gaussian kernel. The resting-state fMRI data were further denoised using the eXtensible Connectivity Pipeline (XCP-engine) software. A high-parameter stream (36p) pipeline was used, combining frame-to-frame motion estimates, mean signals from white matter and cerebrospinal fluid and quadratic and derivative expansions of these signals (Power et al. 2014; Satterthwaite et al. 2013), as they were outputted during fMRIPrep preprocessing. The data were also despiked, temporally filtered (0.01–0.08 Hz), and spatially smoothed with a 6 mm FWHM isotropic Gaussian kernel.

#### General linear modeling: group analysis of the interval recognition task

The analysis was performed using SPM12 (Functional Imaging Laboratory, UCL, UK) running under Matlab R2020b (The Mathworks, Inc., Natick, MA, USA). For each subject, a General Linear Model (GLM) was estimated, contrasting the listening conditions (both successive and simultaneous presentation of sound stimuli) versus the response screen. For the analysis, the first four volumes were discarded. In addition, confound regressors modelling FD per volume (Power et al. 2014), realignment parameters (translation and rotation) and the first six anatomical CompCor components were included as regressors of no interest in the individual GLMs. Each of the listening events was coded as an event with zero duration and convolved with a canonical hemodynamic response function. Finally, a high pass filter of 128 s was used for the data and first-order autoregression allowed for estimation of temporal autocorrelations*.* We used a contrast of listening versus response to allow for the localization of a task-relevant network underlying auditory perception of intervals. We acknowledge that this contrast captures a variety of processes, including pitch perception, interval encoding, maintenance and mental manipulation of the perceived intervals aided by working memory, comparison of the perceived intervals with pre-existing representations/templates of intervallic relationships and labeling/naming the interval. Thus, the brain regions identified by this contrast are not considered exhaustive to intervallic processing. At the group level, we used a one-sample *t* test to test for significant clusters during interval perception.

#### Regions of Interest (ROI) definition

Based on the group level GLM results, we identified the regions involved in interval perception at a threshold of *p* < 0.001 with a Family Wise Error (FWE) clusterwise correction of *p* < 0.05. In addition, a cluster size limit of 45 voxels was applied. For each of the identified ROIs, following the methodological approach of a variety of studies looking into task-informed resting-state fMRI activity (Lumaca et al. 2019; Ramot et al. 2019; Tian et al. 2007; Ventura-Campos et al. 2013; Yuan et al. 2018), a sphere was created using the MarsBaR toolbox for SPM (Oréfice, Oréfice, Costa, Calucci, and Filho, 2016). The center of the sphere was set at the peak MNI coordinate of each cluster and a 5 mm radius was used.

#### Resting-state time-series extraction

The Rex toolbox (region-of-interest extraction tool; The Gabrieli Lab, MIT; http://www.alfnie.com/software) was used to extract the time-series of the resting-state data from within the above defined ROIs for each participant. The extraction was done in units of percent signal change referenced to the mean value of each ROI (Left Superior Temporal Gyrus, Right Superior Temporal Gyrus, Left Putamen, Left Supramarginal Gyrus, ventromedial Prefrontal Cortex). For each participant a 5 × 5 weighted undirected correlation matrix was created using Pearson’s correlation coefficient in R (R Core Team 2021).

#### Graph theory analysis

To characterize and compare the auditory network across all subjects, we utilized graph-theory measures. To do so, we used BRain analysis using GraPH (BRAPH) theory (Mijalkov et al. 2017), a toolbox written in Matlab that uses the Brain Connectivity Toolbox codebase (https://sites.google.com/site/bctnet/; Rubinov and Sporns 2010) to calculate network matrices. The correlation matrices are based on *r* correlation values utilized in the calculation of two global measures. In this framework, nodes are the spheres created corresponding to peak activations in the task-relevant brain regions. The edges represent the correlations between the temporal activation of pairs of these brain regions. The correlation matrix of each participant is a weighted undirected matrix, where the edges indicate the strength of the connection. This way the information of the strength of the connectivity between all nodes is preserved, as the edge weight is a function of the correlation coefficient of the timeseries between two nodes. This way, both stronger and weaker connections are represented in the graph and contribute accordingly to the computation of the graph measures. The absolute values of all correlations (both positive and negative ones) were used in the calculation of the metrics.

We computed two global measures, namely, average strength and global efficiency. Network strength was used to characterize how strongly the nodes are connected. The network strength on the nodal level is defined as the sum of the weights of all edges connected to a node. The global network strength was calculated as the average of the strengths of all five nodes. Global efficiency was used to characterize information transmission among the nodes of the network. Global efficiency at the nodal level defines the efficiency of the information transfer from one region to the whole network, which assesses the average inverse shortest path length between one node and all other nodes in the network. Global efficiency at the global level, the indicator further used here, is then the average of the global efficiency of all nodes in the graph and is inversely related to the characteristic path length (Latora and Marchiori 2001).

Statistical significance testing was done by extracting the values of the two graph measures for each subject from BRAPH, square-root transforming them to deal with non-normal distribution, and then testing for a group difference using a two-sample *t* test in JASP Team (2023).

#### Correlations between graph measures and behavior

To establish a connection between graph measures and behavioral performance, individuals’ network strength and global efficiency were correlated with their performance in (a) the general music expertise score of the BGS, (b) the Intervals and Scales score of the BGS, (c) the interval recognition task, and (d) the reaction times of the interval recognition task, using Pearson’s coefficient in the first two cases, and Spearman’s rho in the latter two as the fMRI performance data shows ceiling effects and the reaction times are not normally distributed. The reported *p* values are False-Discovery Rate (FDR) corrected for multiple comparisons using the online tool (https://www.sdmproject.com/utilities/?show=FDR).

#### Additional analysis

Although the defined ROIs were based on the voxels of peak activation within each cluster which are located almost exclusively on the left hemisphere, the clusters of activation extend rather symmetrically in both hemispheres. Therefore, we conducted an additional analysis to account for the lateralization of the ROIs and to assess whether contribution of relevant brain regions has been missed in the main analysis. Symmetrical ROIs were created around the peak activation voxels (flipping the sign on the x dimension), and the combined activation clusters were used as a mask to ensure that these ROIs lay within it at least by 90% (for the exact coordinates see Table 2 of Supplementary Information). The time-series of the resting-state data were extracted from these 10 ROIs and, as before, weighted undirected correlation matrices were created using Pearson’s correlation coefficient for each participant. The two global measures, average strength and global efficiency were computed as in the main analysis and group differences were estimated using a two-sample *t* test. Furthermore, individuals’ network strength and global efficiency were correlated with their performance in (a) the general music expertise score of the BGS, (b) the Intervals and Scales score of the BGS, and (c) the interval recognition task, using Pearson’s correlation coefficient in the first two cases, and Spearman’s rho in the latter as the fMRI performance data shows ceiling effects.

**Table 2:** Brain regions activated during listening in the fMRI interval recognition task, together with cluster sizes and peak MNI coordinates. Significant clusters were identified at a threshold of *p* < 0.001 with a Family Wise Error (FWE) clusterwise correction of* p* < 0.05 and cluster size of *k* > 45 voxels

Cluster Name	Size	Peak MNI Coordinates
Right superior temporal gyrus (STG), *posterior division*	1019 voxels	*x* = 60, *y* = – 40, *z* = 12
Left superior temporal gyrus (STG), *posterior division*	292 voxels	*x* = – 67, *y* = – 16, *z* = 4
Ventromedial prefrontal cortex (vmPFC)	153 voxels	*x* = – 1, *y* = 48, *z* = -10
Left putamen	112 voxels	*x* = – 22, *y* = 12, *z* = 4
Left supramarginal gyrus (SMG)	68 voxels	*x* = – 61, *y* = – 46, *z* = 26

#### Control analysis

To ensure that any group differences observed in the graph measures would be specific to the auditory network involved in interval recognition and that any relation between the graph measures and behavior would be ascribed to the relevance of this network for behavioral performance, we conducted a control analysis in two other, well-established resting-state networks, namely, the default mode network (DMN) and the executive control network (EN), where we also checked for group differences in graph measures and correlations between those measures with the behavioral ones. Following the publication of De Pisapia et al., we chose seven regions representative of the DMN and six regions for the EN (De Pisapia et al. 2016; see Table2, supplementary material, for details). The procedure of the analysis is identical with the one described above: spheres of 5 mm radius were constructed centered on the peak MNI coordinates of the network regions, the time-series of the resting-state data from these ROIs were extracted for each participant, a weighted undirected correlation matrix for each network was created using Pearson’s correlation coefficient, the two global measures, average strength and global efficiency were computed and again square-root transformed. Statistical testing for group differences was estimated using a two-sample *t* test and individuals’ network strength and global efficiency were correlated with their performance in (a) the general music expertise score of the BGS, (b) the Intervals and Scales score of the BGS, and (c) the interval recognition task, using Pearson’ s correlation coefficient in the first two cases, and Spearman’s rho in the latter as the fMRI performance data shows ceiling effects.

## Results

### Behavioral results

#### Berlin Gehoerbildung Scale (BGS)

**Fig. 1:**
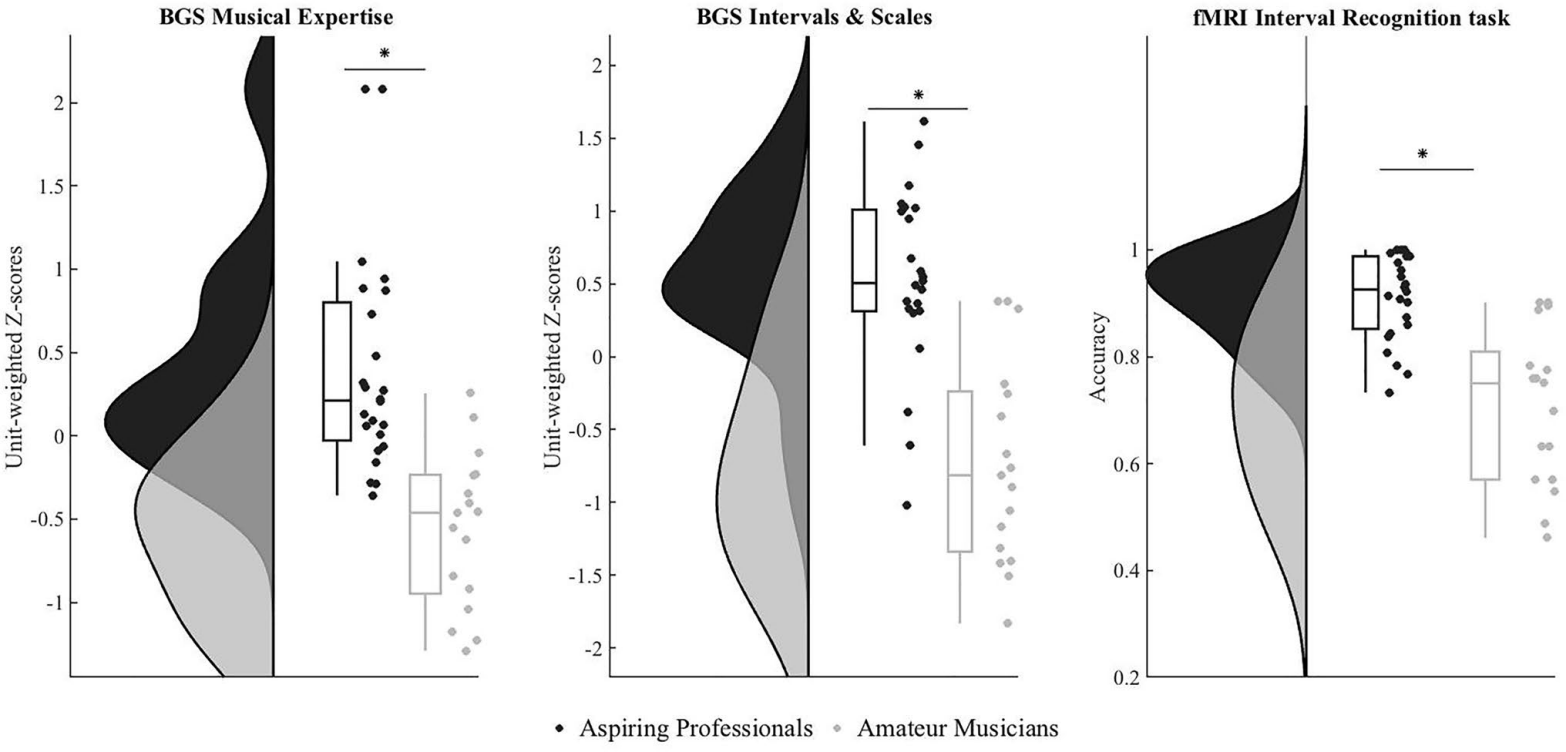
Behavioral performance scores on the Berlin Gehoerbildung Scale (BGS) and the fMRI interval recognition task. In all measures, there was a significant group effect in performance, with aspiring professionals (shown in black) showing higher performance than amateur musicians (in grey), as expected. Group distributions are shown as unmirrored violin plots and boxplots with medians and 95% CI with whiskers representing second and 98th percentiles (Allen et al. 2019). Each dot represents a single subject. Asterisks indicate a significant group effect at *p* < 0.001

As reported before (Wenger et al. 2021), behavioral performance scores on the BGS showed a significant group effect: two-sample *t* tests with the unit-weighted z-scores showed significantly higher levels of performance for aspiring professional musicians compared to amateur musicians on the overall score of music expertise,* t*(39) = 5.72, *p* < 0.001, Cohen’s *d* = 1.8 (amateur musicians *M* = – 0.56, SD = 0.46; aspiring professional musicians* M* = 0.4, *SD* = 0.65), and also on the more specific score of “Intervals and Scales”, *t*(39) = 6.18, *p* < 0.001, Cohen’s *d* = 1.9 (amateur musicians *M* = – 0.74, SD = 0.7; aspiring professional musicians* M* = 0.52, SD = 0.6), see Fig. 1. Of note, there were two extreme cases that were two but not three SDs away from the mean; these were, therefore, not considered outliers but were kept in all further analyses. Importantly, though, the group difference also stayed significant even without them (*t*(37) = 5.686, *p* < 0.001, Cohen’s *d* = 1.64).

#### fMRI Interval recognition task

As the behavioral performance data of the fMRI interval recognition task was not normally distributed but showed a ceiling effect, we first square-root transformed it and then used the Mann–Whitney *U* test for independent samples to non-parametrically analyze group differences in task accuracy (i.e., percentage of correct responses) between aspiring professionals and amateurs. As in the data of the BGS, there was a significant group effect on task accuracy in the fMRI interval recognition task (Mann–Whitney = 40.5, *p* < 0.001, Cohen’s *d* = 4.5). As expected, aspiring professionals (*M* = 83.6, SD = 14.4) exhibited higher accuracy in the task than amateur musicians (*M* = 51.9, SD = 20.5); see Fig. 1. There was also a significant group difference in reaction times with aspiring professionals responding faster than amateur musicians (Mann–Whitney = 292, *p* = 0.02, Cohen’s *d* = 4; aspiring professionals *M* = 3, SD = 1.5, amateur musicians *M* = 4.25, SD = 1.8).

### fMRI task results

**Fig. 2:**
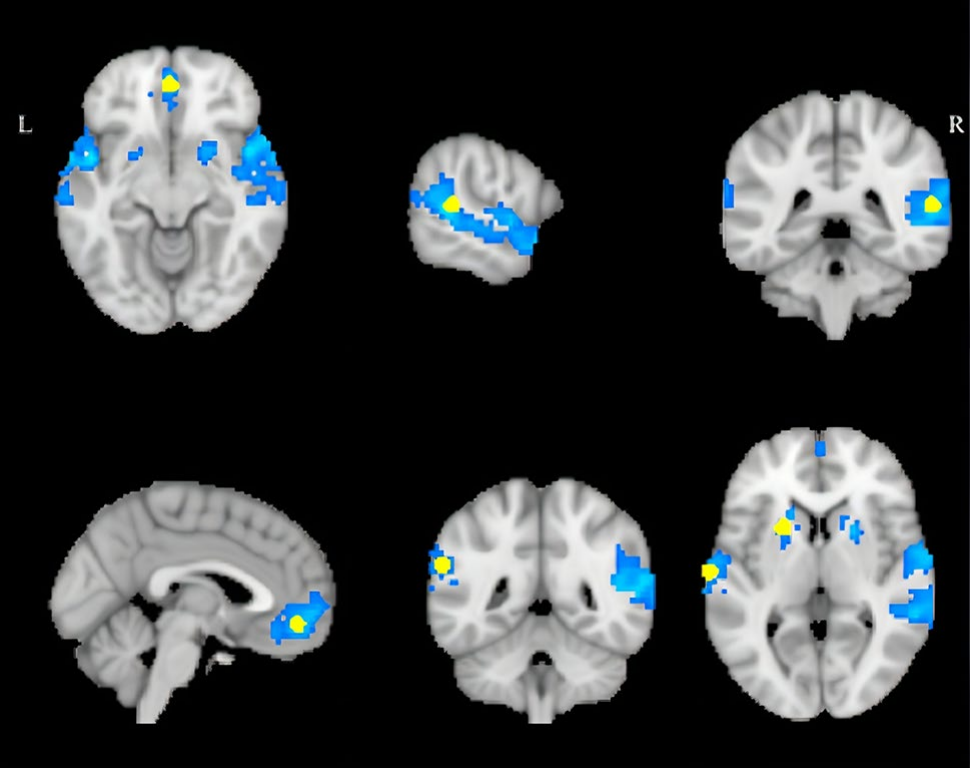
Significant clusters in left and right superior temporal gyrus, ventromedial prefrontal cortex, left putamen and left supramarginal gyrus showing higher activation during listening versus response (*p* < 0.001, clusterwise FWE corrected at *p* < 0.05, cluster size *k* > 45 voxels). Overlaid on the clusters are the spherical ROIs (in yellow) created around the MNI coordinates of peak activation voxels within the clusters

A whole-brain analysis examining the effects of listening versus response across all participants indicated higher activation during the listening condition in the following clusters: left and right superior temporal gyrus (STG) extending both anteriorly and posteriorly bilaterally, including parts of the planum polare, the middle temporal gyrus and the right temporal pole, ventromedial prefrontal cortex (vmPFC), left putamen and left supramarginal gyrus (SMG) (see Table 2 and Fig. 2). As can be seen in Fig. 2, the cluster in the right hemisphere is rather large and extends also into right putamen. However, due to the thresholds used and the loci of peak activation within the cluster, right putamen did not constitute a separate cluster of activation. Rather, left and right STG, vmPFC as well as left putamen and left SMG were considered the network underlying interval recognition and were used as reference points in the creation of ROIs.

### fMRI resting-state graph theoretical analysis

**Fig. 3:**
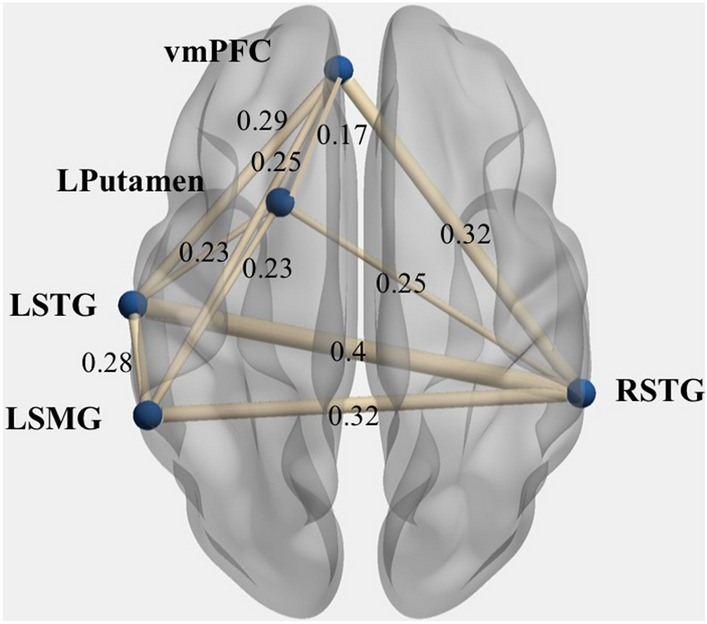
Auditory network as identified based on the interval recognition task and its average correlation between each of the regions for all participants. *LSTG* Left superior temporal gyrus, *RSTG* Right superior temporal gyrus, *LPutamen* Left putamen, *LSMG* Left supramarginal gyrus, *vmPFC* ventromedial prefrontal cortex). Displayed are also the pairwise correlation coefficients between each pair of nodes (uncorrected). The brain networks were visualized with the BrainNet Viewer (http://www.nitrc.org/projects/bnv/), (Xie et al. 2013)

Using spheres built around the peak coordinates of the regions identified in the interval recognition task GLM, we went on to examine activity and connectivity in those regions in the resting-state data. First, the correlations of the extracted time series between each region of the network to the remaining four regions were investigated. The average correlation matrix, rendered as a network, provides information about the average structure of the functional network across all 41 participants (Fig. 3). To characterize the network for each participant in terms of connection strength and efficiency in information transmission and to compare the two groups, graph theory was used and the graph measures of network strength and global efficiency were calculated.

**Fig. 4:**
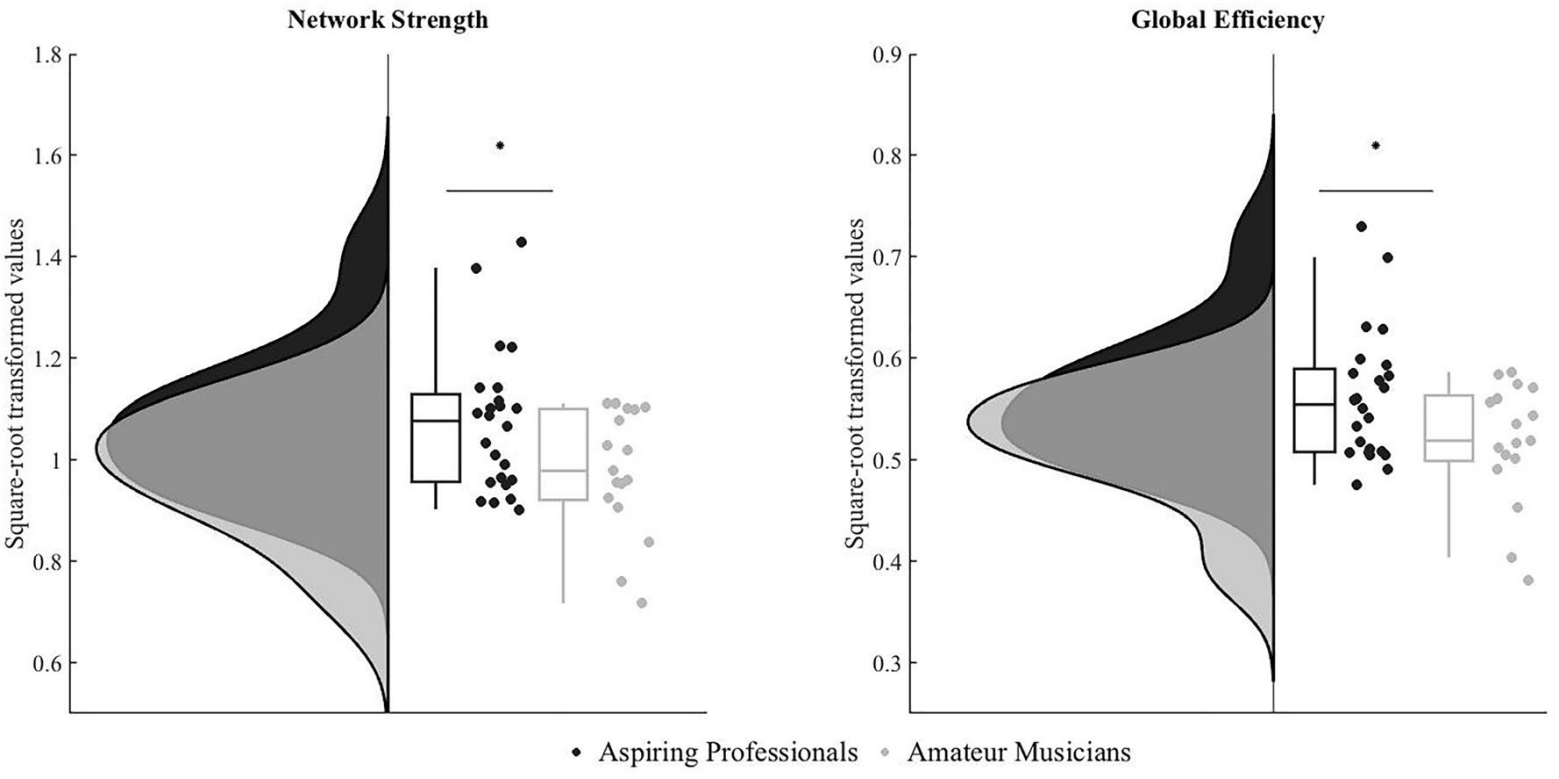
Group comparisons of graph measures network strength and global efficiency. The group of aspiring professionals (in black) showed greater average network strength and global efficiency than amateur musicians (in grey). Group distributions are shown as unmirrored violin plots and boxplots with medians and 95% CI with whiskers representing second and 98th percentiles. Each dot represents a single subject. Asterisks indicate a significant group effect at *p* < 0.05

The average network strength and global efficiency was compared between the two groups using two-sample *t* tests. Aspiring professional musicians indeed showed significantly greater network strength (*t*(39) = 2.213, *p* = 0.03, Cohen’s *d* = 0.7; amateur musicians *M* = 0.97, SD = 0.12; aspiring professional musicians* M* = 1.07, SD = 1.13) and global efficiency (*t*(39) = 2.235, *p* = 0.03, *Cohen’s d* = 0.7; amateur musicians *M* = 0.51, SD = 0.05; aspiring professional musicians* M* = 0.56, SD = 0.06) than amateur musicians (Fig. 4).

### Correlations between graph-theory measures and behavioral performance

**Fig. 5:**
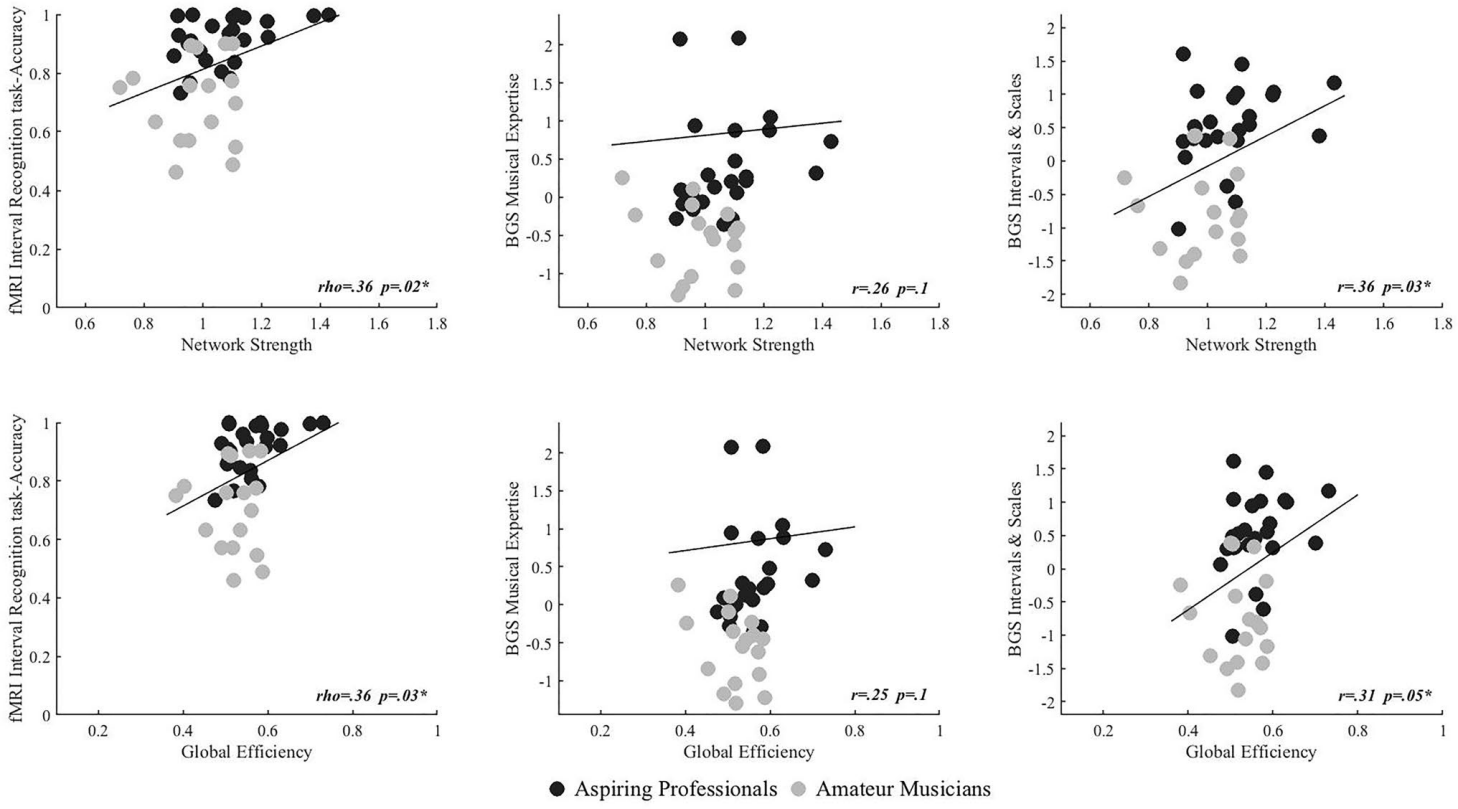
Correlations between graph measures and behavioral performance. Network strength (upper row) and global efficiency (lower row) correlated positively with accuracy in the fMRI interval recognition task (both across groups and within aspiring professionals only) and the BGS “Intervals and Scales” factor, but not with overall BGS “Musical Expertise”. Asterisks indicate significant correlations following FDR correction

The Spearman’s *rho* correlation coefficient between each individual’s network strength on one hand and accuracy in the fMRI intervals recognition task on the other hand revealed a significant positive correlation (*ρ* = 0.36 *p*_FDR_ = 0.02). Likewise, we found a positive correlation between network strength and the BGS “Intervals and Scales” scores (*r* = 0.35 *p*_FDR_ = 0.03), but not with the BGS Musical Expertise scores (*r* = 0.26, *p*_FDR_ = 0.1), see Fig. 5. In addition, we found a significant positive correlation between global efficiency and accuracy in the fMRI intervals recognition task (*rho* = 0.33, *p*_FDR_ = 0.03), with the BGS “Intervals and Scales” scores (*r* = 0.31, *p*_FDR_ = 0.04), but not with the BGS Musical Expertise scores (*r* = 0.25, *p*_FDR_ = 0.1; see Fig. 5). There were no significant correlations between graph measures and reaction times in the fMRI intervals recognition task.

### Additional analysis

As the clusters of activation extend in both hemispheres in a rather symmetrical fashion, while the coordinates of voxels of peak activation around which the ROIs were built lie almost exclusively in the left hemisphere (except the right STG), we also conducted the same line of analysis in a network comprising of these 5 ROIs and their mirror-flipped ROIs. As in the main analysis, aspiring professional musicians showed significantly greater network strength (*t*(39) = 2.34, *p* = 0.02, Cohen’s* d* = 0.75; amateur musicians *M* = 2.4, SD = 0.36; aspiring professional musicians* M* = 2.7, SD = 0.36) and global efficiency (*t*(39) = 2.58, *p* = 0.01, Cohen’s* d* = 0.82; amateur musicians *M* = 0.3, SD = 0.04; aspiring professional musicians* M* = 0.34, SD = 0.03) than amateur musicians. However, in this additional analysis, correlations between network strength and behavioral performance (*ρ* = 0.21, *p* = 0.1 for fMRI Interval Recognition task, *r* = 0.08, *p* = 0.6 for the BGS Musical Expertise and *r* = 0.17, *p* = 0.1 for BGS Intervals and Scales) and also between global efficiency and behavioral performance (*ρ* = 0.25, *p* = 0.1 for fMRI Interval Recognition task, *r* = 0.1, *p* = 0.5 for the BGS Musical Expertise and *r* = 0.2, *p* = 0.2 for BGS Intervals and Scales) failed to reach significance. Given our rather small sample size, it is not surprising that correlations between graph measures and behavioral indices do not hold unequivocally across different network definitions.

### Control analysis

We also compared average network strength and global efficiency between the two groups in the typical DMN and EN using two-sample *t* tests. Professional musicians and amateur musicians did not differ in terms of network strength in the DMN (*t*(39) = 0.413, *p* = 0.7, Cohen’s* d* = – 0.131) or the EN (*t*(39) = 0.152, *p* = 0.8, Cohen’s* d* = – 0.048), nor in terms of global efficiency in the DMN (*t*(39) = 0.580, *p* = 0.6, Cohen’s* d* = – 0.184) or the EN (*t*(39) = 0.6, *p* = 0.6, Cohen’s* d* = – 0.191). There were no significant correlations between DMN network strength and behavioral performance (Cohen’s = 0.12, *p* = 0.4 for fMRI Interval Recognition task, *r* = 0.08, *p* = 0.6 for the BGS Musical Expertise and *r* = 0.05, *p* = 0.7 for BGS Intervals and Scales). There were also no significant correlations between global efficiency and behavioral performance (Cohen’s = 0.12, *p* = 0.4 for fMRI Interval Recognition task, *r* = 0.07, *p* = 0.6 for the BGS Musical Expertise and *r* = 0.09, *p* = 0.5 for BGS Intervals and Scales). Similarly, there were no significant correlations between EN network strength and behavioral performance (*Cohen’s* = 0.14, *p* = 0.3 for fMRI Interval Recognition task, *r* = 0.07, *p* = 0.7 for the BGS Musical Expertise and *r* = – 0.03, *p* = 0.8 for BGS Intervals and Scales), nor between EN global efficiency and behavioral performance (Cohen’s = 0.2, *p* = 0.2 for fMRI Interval Recognition task, *r* = 0.08 *p* = 0.5 for the BGS Musical Expertise and *r* = 0.04, *p* = 0.8 for BGS Intervals and Scales).

## Discussion

In this study, we used data of aspiring professional and amateur musicians, who completed a behavioral test on music expertise called Berlin Gehoerbildung Scale (BGS), as well as an fMRI interval recognition task and an fMRI resting-state scan. We investigated the relationship between resting-state graph measures of an auditory network with behavioral performance. We first used the fMRI interval recognition task and defined an auditory network of regions activated during listening, eventually consisting of left and right superior temporal gyrus (STG), ventromedial prefrontal cortex (vmPFC), left putamen and left supramarginal gyrus (SMG). We then used resting-state fMRI to assess the functional connectivity of those regions, where network strength and global efficiency differed significantly between the two groups. Moreover, network strength as well as global efficiency were significantly associated with behavioral performance in the fMRI task and network strength was as well-associated with the measure of Intervals and Scales of the BGS, but not with the BGS measure of musical expertise. These group differences as well as the correlations between graph measures and behavioral measures were specific to the auditory network involved in interval recognition, and did not occur within the typical default mode or executive control network.

The two largest clusters of activation reported from the fMRI task lie on the *left and right auditory STG*, extending in both hemispheres in the posterior and anterior parts including also parts of the right *Middle Temporal Gyrus (MTG)* with peak activation in *posterior STG* bilaterally, *Planum Polare* bilaterally and the *right Temporal Pole.* Regions within these clusters correspond to the primary auditory cortices as well as belt and parabelt regions which constitute the secondary associative auditory cortices. Activations in the reported regions are in line with the most prevalent findings in studies regarding various aspects of tonal and general auditory processing, typically with a rightward hemispheric functional asymmetry, as right STG appears more specialized for spectral features processing, while the left STG is more specialized for temporal feature processing (Zatorre and Belin 2001). Brain regions, such as the Heschl’s gyrus and adjacent surfaces have been functionally related to auditory pitch perception, while pitch changes have been related to activation in the right STG and additionally in right planum temporale and planum polare and anterior parts of the STG (Hyde et al. 2008; Patterson et al. 2002; Warren and Griffiths 2003). The right posterior STG is reported in addition to play a role in imagery or rehearsal of tones and melodies (Peretz and Zatorre 2005), auditory working memory (Nolden et al. 2013), and perceptual decision making (King et al. 2018; McDermott and Oxenham 2008). Overall, interval information processing appears to involve areas anterior and posterior of the supratemporal plane (Koelsch 2011), where also our clusters of activation extend.

Apart from the superior temporal areas, three additional clusters were found in extra-auditory regions in the basal ganglia, the medial orbitofrontal cortex and the left supramarginal gyrus. The *left and right putamen*, parts of the dorsal striatum, are related to a wide-range of functions from sensorimotor to decision making and reward processing (Groenewegen 2003). In relation to audition, evidence from animal studies has established the role of corticostriatal neurons in auditory decisions (Znamenskiy and Zador 2013) and in integration of multisensory information (Zhong et al. 2014). In humans, putamen activation has been detected in a variety of auditory processes, including beat perception, sensory-motor predictability, finger tapping, music comprehension, tone discrimination, audiomotor coupling assumed to relate to temporal and sequential aspects of processing (i.e., syntax in language) and musical imagery (Geiser et al. 2012; Kotz et al. 2009; Pando-Naude et al. 2021). The *left SMG*, part of the somatosensory association cortex, apart from its involvement in phonological and articulatory processes (Oberhuber et al. 2016), has been shown to facilitate short-term pitch memory (Schaal et al. 2017; Vines et al. 2006) and maintenance of pitch information in studies using transcranial magnetic stimulation (TMS; Schaal et al. 2015). The *ventromedial prefrontal cortex (vmPFC),* a region receiving projections from multiple sensory areas and limbic structures, plays a central role in sensory-input integration and in perception-based decision-making (Sharma and Bandyopadhyay 2020). Animal studies have shown orbitofrontal activation in response to sound and an association of the orbitofrontal cortex, constituting part of the vmPFC, with the primary auditory cortex (Winkowski et al. 2013, 2018). In humans, activation of the vmPFC and ventrolateral PFC has been reported during auditory processes, involving attending to pitch, rhythm and melodies, determining sound length and auditory working memory (Plakke and Romanski 2014). More importantly, the rostromedial prefrontal cortex has been reported to maintain a topographic representation of the tonality surface (Janata et al. 2002). These findings highlight the role of the medial PFC in maintaining tonal contexts and facilitating integration of information necessary for interval perception and identification.

Consequently, all five regions of the reported network involved in interval recognition have already been associated with various aspects of auditory processing pertinent to the current study in existing literature. We consider pitch and interval processing to be reflected in activation primarily in bilateral STG, short-term maintenance of the auditory information in the left SMG, and integration of information as well as preparation for decision and response in the putamen and vmPFC. Thus, the activation of extra-auditory regions comes as no surprise as these structures mediate different aspects of auditory processing. There exists a rich literature especially regarding the connection between auditory cortex and frontal regions often termed the ventral and dorsal dual stream of auditory processing, in which we suspect our findings to reflect the ventral stream, originating in the primary auditory cortex and projecting to the ventral regions of the frontal cortex (Zulfiqar et al. 2020).

Although a first view on the spherical ROIs created around the voxels with peak activation values gives an impression of general left lateralization of the regions, this does not portray entirely the outcome of the fMRI task analysis. Apart from the left SMG, the clusters of activation were bilateral, as can be seen in Fig. 2. The proximity of activation and the size of the smoothing kernel influenced the formation and the extent of the clusters. Under these restraints, the right putamen belonged to the larger cluster extending onto the right STG and the cluster formed bilaterally on the vmPFC was restricted to the left hemisphere, where the peak activation value of the cluster was located. Furthermore, in additional analysis conducted including apart from those five ROIs their contralateral mirror ROIs, the group differences in graph measures persisted, suggesting that contributions of other regions within the clusters might be missed in the chosen main analysis. Moreover, we did not take into account task-specific demands and task-difficulty for the purposes of this study, which have been pointed out in other studies to impact the lateralization of the observed activity (Angenstein et al. 2012; Brechmann and Angenstein 2019). We, therefore, would like to refrain from making any inferences regarding lateralization of activity.

The group difference in performance in the behavioral task of BGS and the performance in the fMRI task, paralleled by group differences in graph measures of network strength and global efficiency, adds to the rich literature of functional and structural reorganization of the brain in relation to musical training of different intensities and aspirations as well as expertise level (Jäncke 2009; Olszewska et al. 2021; Schlaug 2008; James et al. 2014; James et al. 2017; Oechslin et al. 2013). Average network strength is computed as the sum of all weights of all edges connected to a node, averaged for all nodes (Maudoux et al. 2012). Thus, the greater network strength observed in the group of aspiring professionals indicates stronger functional connectivity among regions of the interval recognition auditory network, irrespective of task execution. Such a finding has already been established using resting-state fMRI, relating musical expertise to increased functional connectivity not only between auditory regions (Luo et al. 2012; Palomar-García et al. 2017; Schlaug 2008) but also between auditory and multisensory and motor regions (Schlaug 2008; Wenger et al. 2021), prefrontal regions (Klein et al. 2016), insular cortex and parietal regions (Luo et al. 2014). Global efficiency, computed as the average of the inverse shortest path length from a node to all others, averaged for all nodes (Latora and Marchiori 2001), points towards more direct and efficient communication between the nodes of a network and functional integration. Therefore, the greater global efficiency observed in the group of aspiring professionals suggests a more efficient information flow and communication between the nodes of an auditory network facilitating interval recognition. Hence, aspiring professionals—either as a result of their training or because of their self-selection based on talent—seem to rely on a more connected and efficient auditory network that underlies their better interval discrimination ability, as suggested by the correlations between the graph measures and behavioral performance. This is also supported by the specificity of the observed group differences in graph measures of the interval recognition network but not the DMN or EN, and the correlations between these graph measures and behavior.

So far, only few studies have applied graph measures to characterize brain networks related to musical training and expertise. One study using a paradigm in which participants listened to music clips reported increased degree, clustering, and local efficiency, especially for the left STG in musicians with absolute pitch compared to musicians without absolute pitch (Loui et al. 2012). Another study using a similar paradigm found significantly higher nodal degree for musicians in cerebellar regions, the right temporal pole, the parahippocampal gyrus and the inferior temporal gyrus (Alluri et al. 2017). In a study where graph measures were applied on whole-brain resting-state fMRI data, musicians had higher average strength, higher clustering coefficient, and, surprisingly, lower global efficiency in comparison with non-musicians (Leipold et al. 2021). In yet another study, however, using resting-state magnetoencephalography (MEG) data, greater global efficiency was reported for musicians, just as we find here (Paraskevopoulos et al. 2017). In a previous study, using the same resting-state fMRI data as the current one and investigating the functional connectivity and graph measures of the left planum polare, which underwent volumetric changes over time, we found that the group of aspiring professionals exhibited significant increases over time in global efficiency and clustering measures (Wenger et al. 2021). This finding speaks in favor of a training-associated, rather than purely talent-based, interpretation of the present results. Still, we do not know whether amateur musicians would have been able to show this change had they been exposed to the exactly identical training environment. Although further research is required to better characterize neural networks underlying auditory processing and musical expertise, we consider the current finding of group differences in graph measures that relate to behavioral outcomes as an important indicator of the potential such approaches have in deepening the understanding of the characteristics of the organization of brain regions underlying specific processes, in relation to different levels of expertise.

The present results also elucidate the relationship between task fMRI and resting-state fMRI. Regions co-activated or exhibiting heightened functional connectivity while executing a specific task are thought to form a task-relevant functional network. During resting-state fMRI, such co-activation of brain regions also occurs and appears organized in several large-scale resting-state networks, reproducible across research institutes and populations (Damoiseaux et al. 2006; van den Heuvel and Hulshoff Pol 2010). One part of these networks is typically also an auditory one, encompassing primarily bilateral primary and associative auditory cortices and often, including other brain regions, such as insula, prefrontal, sensorimotor, anterior cingulate and left occipital cortices (Maudoux et al. 2012). A series of studies and an impressive meta-analysis of a large number of fMRI studies have shown that task-related activation patterns can indeed be mapped onto resting-state networks (Calhoun et al. 2008; Cole et al. 2014, 2016; Di et al. 2013; Simon-Vermot et al. 2018; Smith et al. 2009). Such findings suggest that regions intrinsically connected during resting state become simultaneously activated during task execution. In addition, individual variability in resting state has been found to be correlated and predictive of individual variability in cognitive and motor tasks (Tavor et al. 2016) as well as in processes of emotional regulation and decision making (Cole et al. 2014, 2016). Such findings have led to a conceptualization of intrinsic network architectures, as captured in resting state, that are further shaped and altered during task execution by specific task demands (Cole et al. 2014, 2016). We consider the results reported in this study to add further to this literature by demonstrating that an auditory network extracted during execution of the specific process of interval recognition, not only retains its functional organization in resting state, but further that graph measures outlining its strength and efficiency can characterize musical expertise and predict behavioral performance.

Finally, we wish to address some limitations of the current study. As the accuracy data of the fMRI interval recognition task was not normally distributed, the interpretation of the significant correlation between task accuracy and network strength and global efficiency should be taken with a grain of salt. Nevertheless, we see a clear tendency of greater network strength associated with better performance not only in the fMRI interval recognition task, but also the “Intervals and Scales” measure of the BGS. Obviously, the current results do not answer the question whether amateur musicians did not recognize some of the different intervals or were simply unable to correctly name them. Still, the correlation between network strength and global efficiency with behavioral performance suggests a link between the more general feature of music expertise (which entails studying of how to correctly name intervals) and brain networks. Future research should try to disentangle differences between correct perceptual recognition of smaller versus greater intervals, and the ability to correctly name them. Furthermore, we would like to highlight that the network of regions reported here, based on the loci of peak activation within each significant cluster from the task-fMRI analysis, is a network facilitating interval perception and recognition, but is not exhaustive in the regions it includes. The contrast of listening versus response does not allow for a very precise localization of tonal processes or for deciphering between simultaneously and sequentially presented intervals. In addition, although the significant clusters of activity are rather extensive, especially along the STG bilaterally, the spherical ROIs cover only a small part of the clusters, making them indicative of the strength of activation in this region but not very fine-grained in their precision. Furthermore, motivation differences between the two groups were not assessed with a standardized measure regarding participants’ motivation in relation to their engagement to music. We consider, however, that differences in motivation between participants in the two groups may be accepted as a given in aspiring professional musicians preparing for an entrance exam to study music*.* Finally, we need to acknowledge the basic limitation that participants were not randomly assigned to the different groups, an issue that often arises when comparing groups with different levels of expertise. The decisive difference between the groups is the professional intention which is also reflected in the intensity of daily training, practical and theoretical, which they undertake. This limitation was attenuated, but not overcome, by matching participants in both groups on years of playing music. Given the pervasive presence of gene-environment correlations (Ullén et al. 2016), it is likely that participants in the two groups differed in their propensity to profit from extended musical practice.

## Conclusion

In this study, a functional network defined on the basis of fMRI activations during interval recognition differed in strength and global efficiency between amateur musicians and aspiring professionals. Furthermore, network strength and global efficiency correlated with performance on the fMRI interval recognition task as well as with the ability to name and identify intervals and scales assessed with the BGS, a psychometrically validated test of musical expertise. Together, these findings highlight how task-informed resting-state fMRI can capture persisting expertise-associated connectivity differences underlying task execution and relate them to expertise-associated behavioral performance. Aspiring professionals, presumably as a result of their training, seem to rely on a more connected and efficient auditory network that supports expert performance levels. The observed group differences in connectivity and global efficiency at rest in a task-relevant network may point to trait-like domain-specific differences in the intensity and efficiency of neural communication.

### Supplementary Information

Below is the link to the electronic supplementary material.

Supplementary file1 (DOCX 21 KB)

Supplementary file2 (DOCX 23 KB)

## Data Availability

The data that support the findings of this study are available from the corresponding author EP upon reasonable request.
