# Supplementary material for: Resting-state functional connectivity in an auditory network differs between aspiring professional and amateur musicians and correlates with performance
Source: Brain Struct Funct. 2023 Oct 4;228(9):2147–63. doi: 10.1007/s00429-023-02711-1 (PMC10587189; doi:10.1007/s00429-023-02711-1)
Supplement: Supplementary file 2 — Supplementary file2 (DOCX 23 KB) [file 429_2023_2711_MOESM2_ESM.docx]

**Resting state functional connectivity in an auditory network differs between aspiring professional and amateur musicians and correlates with performance**

Eleftheria Papadaki^1,2*^, Theodoros Koustakas^1^, André Werner^1^, Ulman Lindenberger^1,3^,

Simone Kühn^4,5^, & Elisabeth Wenger^1^

^1^ Center for Lifespan Psychology, Max Planck Institute for Human Development, Berlin, Germany

^2^ International Max Planck Research School on the Life Course (LIFE), Berlin, Germany

^3^ Max Planck UCL Centre for Computational Psychiatry and Ageing Research, Berlin, Germany, and London, UK

^4^ Lise Meitner Group for Environmental Neuroscience, Max Planck Institute for Human Development, Berlin, Germany

^5^ Neuronal Plasticity Working Group, Department of Psychiatry and Psychotherapy, University Medical Center Hamburg-Eppendorf, Hamburg, Germany

*** Corresponding author:**

Eleftheria Papadaki, Center for Lifespan Psychology, Max Planck Institute for Human Development, Lentzeallee 94, 14195 Berlin, Germany; Email: [papadaki@mpib-berlin.mpg.de](file:///Users/papadaki/Library/Containers/com.microsoft.Word/Data/Downloads/papadaki@mpib-berlin.mpg.de)

Journal: Brain Structure and Function

Supplementary Information

| Primary music instrument | | Aspiring professional musicians | Amateur musicians |
| --- | --- | --- | --- |
| String instruments | struck (piano) | 6 | 3 |
|  | bowed (violin,cello) | 5 | 4 |
|  | plucked (gitarre) | 3 | 6 |
| Percussion | | 3 | - |
| Wind instruments (trompete, saxophone,flute) | | 4 | 4 |
| Singing | | 2 | - |

**Table 1.** Primary musical instruments reported by participants in both groups and distributed according to the type of instrument (string instruments, percussion, wind instruments, voice).

| **Regions of interest (ROIS)** | **MNI Coordinates** | | |
| --- | --- | --- | --- |
|  | X | Y | Z |
| right superior temporal gyrus, *posterior division* | 60 | -40 | 12 |
| left superior temporal gyrus, *posterior division* | -67 | -16, | 4 |
| ventromedial prefrontal cortex | -1 | 48 | -10 |
| left putamen | -22 | 12 | 4 |
| left supramarginal gyrus | -61 | -46 | 26 |
| left superior temporal gyrus, *posterior division* (mirror ROI) | -60 | -40 | 12 |
| right superior temporal gyrus, *posterior division* (mirror ROI) | 67 | -16, | 4 |
| ventromedial prefrontal cortex (mirror ROI) | 1 | 48 | -10 |
| right putamen (mirror ROI) | 22 | 12 | 4 |
| right supramarginal gyrus (mirror ROI) | 61 | -46 | 26 |

**Table 2.** Regions of interest with coordinates in MNI space used in an additional analysis to account for the left lateralization of the ROIs of the main analysis.

| **Regions** | **MNI coordinates** | | | **Network** |
| --- | --- | --- | --- | --- |
|  | **X** | **Y** | **Z** |  |
| Medial Prefrontal Cortex | -1 | 49 | -5 | DMN |
| Posterior Cingulate Cortex | -6 | -52 | 40 | DMN |
| Precuneus | 0 | -56 | 28 | DMN |
| Left Precuneus/Posterior Cingulate Cortex | -10 | -66 | 24 | DMN |
| Right Precuneus/Posterior Cingulate Cortex | 10 | -66 | 24 | DMN |
| Left Lateral Parietal | -46 | -70 | 36 | DMN |
| Right Lateral Parietal | 46 | -70 | 36 | DMN |
| Left anterior dorsolateral Prefrontal Cortex | -27 | 63 | 6 | EN |
| Right anterior dorsolateral Prefrontal Cortex | 27 | 63 | 6 | EN |
| Left dorsolateral prefrontal cortex | -46 | 38 | 12 | EN |
| Right dorsolateral prefrontal cortex | 46 | 38 | 12 | EN |
| Left Inferior Frontal Gyrus | -40 | 24 | -10 | EN |
| Right Inferior Frontal Gyrus | 40 | 24 | -10 | EN |

**Table 3.** Brain regions of the default mode network (DMN) and the executive control network (EN) with coordinates in MNI space (see De Pisapia et al., 2016) used in an additional control analysis to test the specificity or generalizability of our current findings.

Reference

De Pisapia, N., Bacci, F., Parrott, D., & Melcher, D. (2016). Brain networks for visual creativity: A functional connectivity study of planning a visual artwork. *Scientific Reports*, *6*, 1–11. https://doi.org/10.1038/srep39185
